# In-vivo cardiac DTI: An initial comparison of M012 compensated spin-echo and STEAM

**DOI:** 10.1186/1532-429X-18-S1-W19

**Published:** 2016-01-27

**Authors:** Andrew D Scott, Sonia Nielles-Vallespin, Pedro Ferreira, Zohya Khalique, Laura-Ann McGill, Philip J Kilner, Dudley J Pennell, David Firmin

**Affiliations:** 1 Cardiovascular Biomedical Research Unit, The Royal Brompton Hospital, London, United Kingdom; 2 National Heart and Lung Institute, Imperial College London, London, United Kingdom; 3 National Heart Lung and Blood Institute, National Institutes for Health, Bethesda, MD USA

## Background

In-vivo cardiac diffusion tensor imaging (cDTI) has been performed using a stimulated echo (STEAM) sequence for 20 years [1]. While short diffusion gradients make it motion insensitive, it is strain sensitive and SNR inefficient. Recently a spin-echo (SE) sequence with velocity and acceleration compensated diffusion gradients was demonstrated in rats [2] and healthy volunteers using high performance gradients [3]. This sequence is insensitive to strain and should have higher SNR than STEAM, but diffusion gradient duration and hence TE is increased while mixing time is decreased. Here we implement a velocity and acceleration compensated SE cDTI sequence on a clinical 3T scanner and show initial comparisons with STEAM.

## Methods

A SE EPI cDTI sequence was implemented with 0^th^, 1^st^ and 2^nd^ order motion-compensated diffusion gradients (M012) [2, 3]. Mid-ventricular short-axis cDTI was performed in 10 healthy volunteers on a 3T Siemens Skyra (Gradients 45 mT/m@200 Tm/s per axis) with both M012 and STEAM [4]. Acquisitions were performed at end-systole, end-diastole and 150 ms from the R-wave (average systolic sweet-spot [5]). Time from R-wave to diffusion encoding was matched between sequences. M012 acquisitions used b_main_ = 450 smm^-2^, TE = 73 ms and water-selective excitation. STEAM acquisitions used b_main_ = 800 smm^-2^, TE = 23 ms and fat saturation. Both acquisitions used 6 diffusion directions, b_ref_ = 150 smm^-2^, 6 averages, TR = 2RR-intervals, reduced phase field-of-view, 360 × 135 × 8 mm^3^ at 2.8 × 2.8 mm^2^ resolution, SENSE x2 and an identical EPI echo train. Each breath-hold was 20RR for both sequences. Since STEAM requires 2RR for diffusion encoding the M012-SE sequence was triggered to alternate R-waves.

## Results

**Figure 1:**
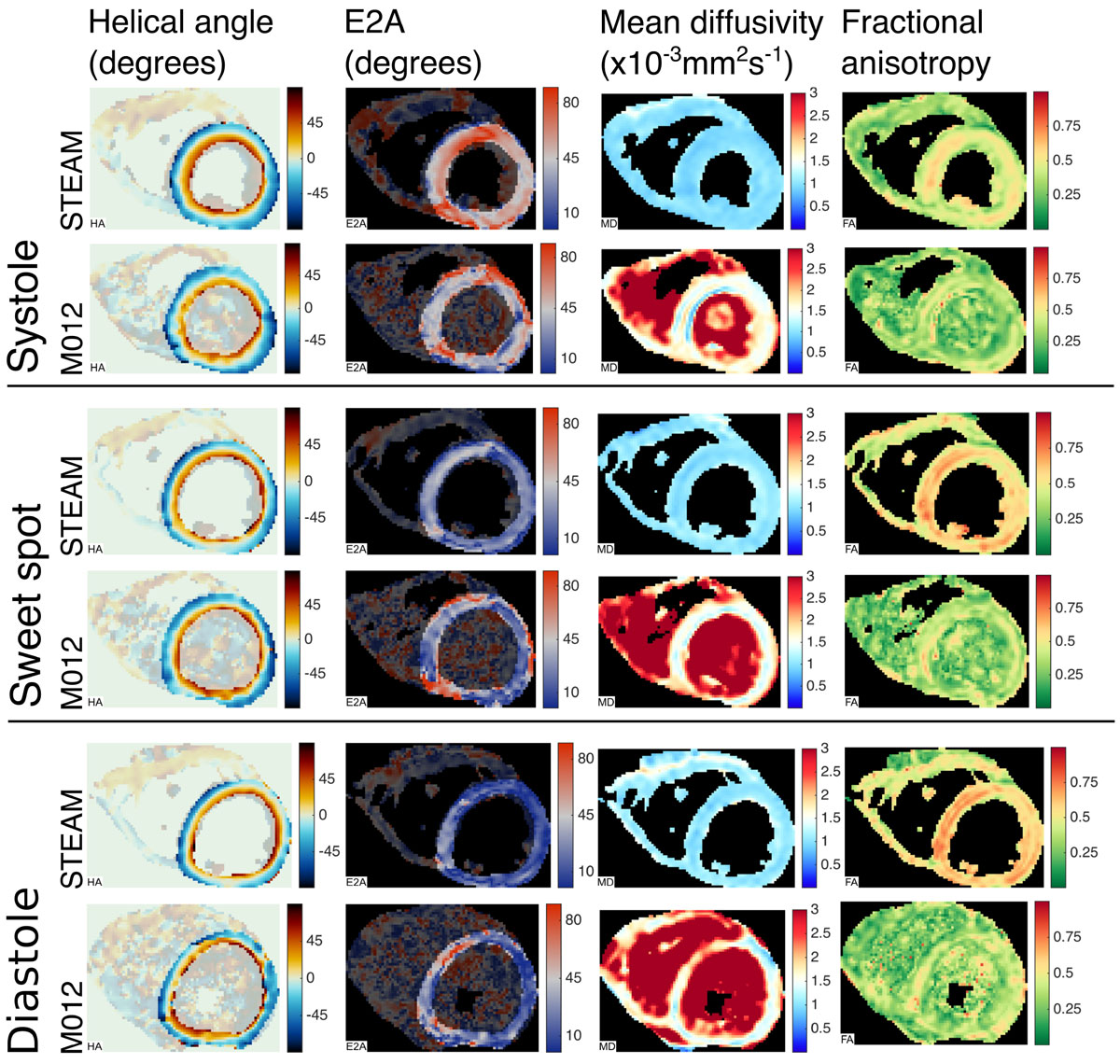
**Parameter maps acquired in one example subject using the STEAM and M012 compensated spin-echo sequences at end-systole, approximate sweet-spot (150 ms from R-wave) and end-diastole**. While helical angle maps appear relatively similar between the two sequences, there are clear differences in the other parameters. Note that, unlike the STEAM sequence the M012 sequence is not intrinsically a dark blood method. The R-wave to diffusion encoding time was matched between sequences to provide the most meaningful comparison between the parameters. As a result there may be slight differences in the shape of the heart between sequences due to the difference in R-wave to imaging time.

**Figure 2:**
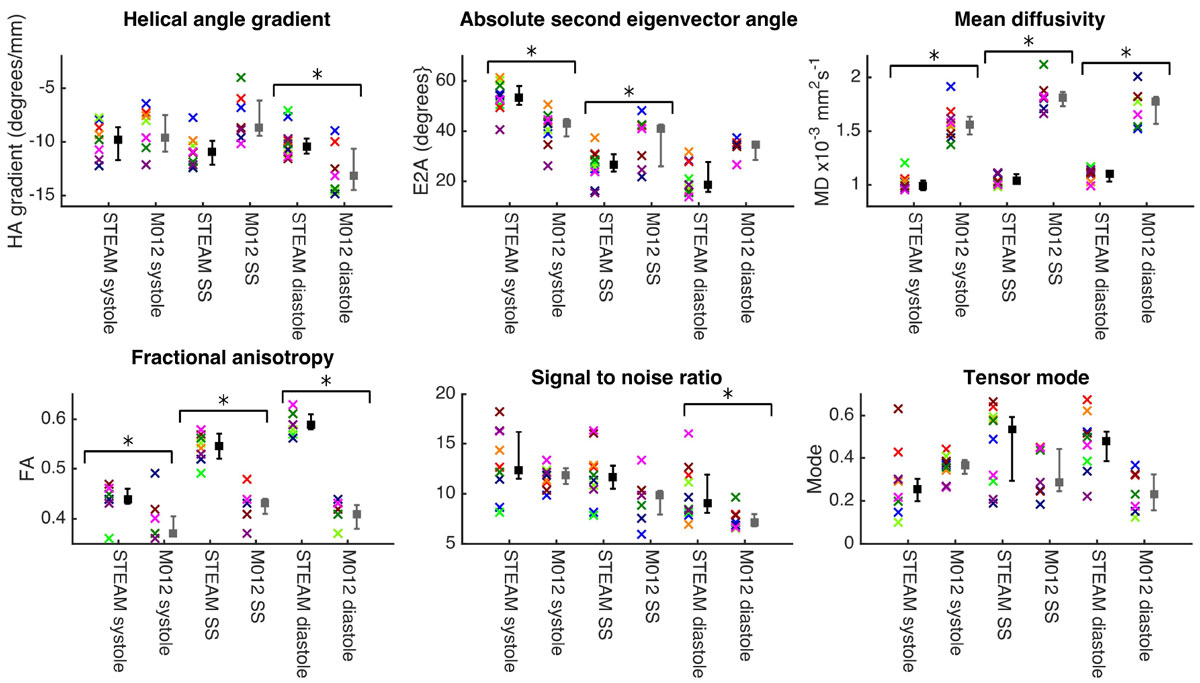
**Parameters derived from cDTI and averaged over the left ventricle plotted for all subjects (crosses coloured by subject), time points and both sequences**. Median, 25^th^ and 75^th^ percentiles are plotted in black for STEAM and grey for M012. Paired comparisons between M012 and STEAM for each parameter were performed at each time point using a Mann-Whitney U-test. Significant comparisons at the p = 0.05 level are indicated with *. Acquisitions deemed unevaluable were excluded from this analysis. SNR was measured in the un-averaged b_ref_ images as described in reference 4.

Figure 1 shows parameter maps from one subject using both sequences at all 3 time points. All STEAM acquisitions were considered evaluable. For M012: 1/10 systolic, 3/10 sweet spot and 3/10 diastolic data sets were not evaluable due to bulk motion related signal loss. Figure 2 compares helical angle gradient, absolute second eigenvector angle (E2A) [6], mean diffusivity (MD), fractional anisotropy (FA) and SNR measured in the left ventricle from all acquisitions. MD is lower and FA is higher using M012 (both p < 0.05). Differences in E2A between systole and diastole are reduced using M012. SNR is higher using STEAM (diastole: p < 0.05).

## Conclusions

M012 compensated SE cDTI can be performed on most subjects at 3T with clinical gradients but its accuracy and reproducibility relative to other techniques requires further evaluation. Preliminary results show that STEAM is more reliable and the expected improvement in SNR using the M012 sequence was not observed. Parameters may vary significantly between techniques due several factors including: T1 and T2-weighting, strain sensitivity, motion sensitivity and mixing time.
